# Verstehen im Störschall mit räumlich getrennten Signalquellen und Richtungshören mit Sprachstimuli: Lerneffekte und Reproduzierbarkeit

**DOI:** 10.1007/s00106-024-01426-x

**Published:** 2024-03-27

**Authors:** Svenja Buth, Izet Baljić, Alexander Mewes, Matthias Hey

**Affiliations:** 1 Medizinische Fakultät, Christian-Albrechts-Universität zu Kiel, Kiel, Deutschland; 2 HNO-Klinik, Audiologie, Campus Kiel, Universitätsklinikum Schleswig-Holstein, Arnold-Heller-Str. 3, Haus B1, 24105 Kiel, Deutschland; 3 Klinik für Hals‑, Nasen‑, Ohrenheilkunde, Audiologisches Zentrum, Helios Klinikum Erfurt, Erfurt, Deutschland; 4 Klinik für Hals‑, Nasen‑, Ohrenheilkunde, Kopf- und Halschirurgie, Audiologie, UKSH, Kiel, Deutschland

**Keywords:** Binaurales Hören, Sprachverstehen im Störschall, Getrennte Signalquellen, Right-Ear-Advantage-Effekt, Richtungshören, Binaural hearing, Speech intelligibility in noise, Separated sound sources, Right-ear advantage, Sound localization

## Abstract

**Hintergrund:**

Binaurales Hören ermöglicht das bessere Sprachverstehen in geräuschvollen Umgebungen und stellt eine Voraussetzung für die akustische Raumorientierung dar. Deshalb soll im Rahmen dieser Studie das Sprachverstehen im Störschall bei separierten Signalquellen und das Richtungshören untersucht werden. Ziel war es dabei, Kennwerte und Reproduzierbarkeiten für zwei ausgewählte Testverfahren, welche für die Beschreibung der beiden genannten Aspekte des binauralen Hörens als geeignet scheinen, zu erheben.

**Methode:**

Bei 55 normalhörenden Erwachsenen wurden die Sprachverständlichkeitsschwellen im Störschall („speech reception thresholds“ [SRT]) und die Test-Retest-Reliabilität bei räumlich getrennten Signalquellen im 45°- und 90°-Winkel für den Oldenburger Satztest erhoben. Die Untersuchung des Richtungshörens erfolgte für den Halb- und Vollkreis (7 und 12 äquidistante Lautsprecher).

**Ergebnisse:**

Es wurden SRT (S_−45_N_45_: −14,1 dB SNR, S_45_N_−45_: −16,4 dB SNR, S_0_N_90_: −13,1 dB SNR, S_0_N_−90_: −13,4 dB SNR) und die Test-Retest-Reliabilität (4 bis 6 dB SNR) für das Sprachverstehen im Störschall bei separierten Schallquellen erhoben. Der prozedurale Lerneffekt konnte erst bei Einsatz von 120 Trainingssätzen minimiert werden. Es wurde eine signifikant niedrigere SRT für die Prüfsituation des rechten Ohrs im Vergleich zum linken ermittelt. Für das Richtungshören im Halbkreis konnten RMS-Werte von (1,9°) und für den Vollkreis von (11,1°) erhoben werden. Hierbei zeigten sich in der Wiederholungsmessung des Vollkreises bessere Ergebnisse.

**Schlussfolgerung:**

Beim Einsatz des Oldenburger Satztests im Störschall mit separierten Signalquellen besteht die Notwendigkeit eines ausgedehnten Trainings mit mehr als 120 Sätzen, um den prozeduralen Lerneffekt zu minimieren. Es sind seitenspezifische SRT-Werte erforderlich, welche vermutlich durch den Right-Ear-Advantage-Effekt bedingt sind. Für die Durchführung des Richtungshörens im Vollkreis wird ein Training empfohlen.

## Abstract (en)

**Background:**

Binaural hearing enables better speech comprehension in noisy environments and is necessary for acoustic spatial orientation. This study investigates speech discrimination in noise with separated signal sources and measures sound localization. The aim was to study characteristics and reproducibility of two selected measurement techniques which seem to be suitable for description of the aforementioned aspects of binaural hearing.

**Materials and methods:**

Speech reception thresholds (SRT) in noise and test–retest reliability were collected from 55 normal-hearing adults for a spatial setup of loudspeakers with angles of ± 45° and ± 90° using the Oldenburg sentence test. The investigations of sound localization were conducted in a semicircle and fullcircle setup (7 and 12 equidistant loudspeakers).

**Results:**

SRT (S_−45_N_45_: −14.1 dB SNR; S_45_N_−45_: −16.4 dB SNR; S_0_N_90_: −13.1 dB SNR; S_0_N_−90_: −13.4 dB SNR) and test–retest reliability (4 to 6 dB SNR) were collected for speech intelligibility in noise with separated signals. The procedural learning effect for this setup could only be mitigated with 120 training sentences. Significantly smaller SRT values, resulting in better speech discrimination, were found for the test situation of the right compared to the left ear. RMS values could be gathered for sound localization in the semicircle (1,9°) as well as in the fullcircle setup (11,1°). Better results were obtained in the retest of the fullcircle setup.

**Conclusion:**

When using the Oldenburg sentence test in noise with spatially separated signals, it is mandatory to perform a training session of 120 sentences in order to minimize the procedural learning effect. Ear-specific SRT values for speech discrimination in noise with separated signal sources are required, which is probably due to the right-ear advantage. A training is recommended for sound localization in the fullcircle setup.

Bei der Kommunikation im Alltag sind Menschen oftmals schwierigen Hörsituationen ausgesetzt, die durch mehrere Schallquellen, Nachhall und Störgeräusche charakterisiert sind. Die zentrale Verarbeitung binauraler Signale ermöglicht die Bewältigung dieser Aufgaben, indem Sprache und Störgeräusch getrennt wahrgenommen werden [24, 25]. Hierzu werden interaurale Laufzeit- und Pegelunterschiede der Signale genutzt [24]. Bei der räumlichen Trennung von Signal und Störschall verbessert sich das Sprachverstehen durch die Verarbeitung binauraler Informationen. Dieses Phänomen wird als „spatial release from masking“ bezeichnet und zeigt den Nutzen des beidohrigen Hörens beim Verstehen von Sprache im Störschall [24, 25]. Zudem können mithilfe binauraler Informationen akustische Signale lokalisiert werden, weshalb das beidohrige Hören auch für das Richtungshören von großer Bedeutung ist [19, 25, 37].

Für die komplexe Aufgabe der Sprachaudiometrie wurden in den letzten Jahrzehnten eine Vielzahl von Testverfahren entwickelt [19, 31]. Für die Prüfung des Sprachverstehens im Störschall werden zur Abbildung der alltäglichen Kommunikation bevorzugt Sätze eingesetzt [31]. Basierend auf dem Ansatz der schwedischen Matrixsätze von Hagermann [13] wurde für die Untersuchung im Störschall der Oldenburger Satztest (OLSA) als deutschsprachiges Pendant entworfen [19, 34]. Ziel war es dabei, ein Verfahren zu konzipieren, das eine steile Diskriminationsfunktion besitzt und über eine hohe Anzahl von Testlisten verfügt, sodass der Test möglichst oft an einer Person eingesetzt werden kann [34]. Matrixtests wie der OLSA besitzen eine feste Satzstruktur, wobei in diesem Fall die Sätze aus jeweils einem Namen, Verb, Zahl, Adjektiv sowie Objekt bestehen. Für jedes Wort gibt es 10 Alternativen, sodass eine Anzahl von theoretisch 100.000 Sätzen generiert werden kann [34]. Die Sätze sind grammatikalisch korrekt, ergeben jedoch semantisch oft keinen Sinn und sind daher nur wenig redundant [34].

Neben den positiven Eigenschaften des OLSA erweist sich der prozedurale Lerneffekt innerhalb einer Testsitzung als eine der methodischen Herausforderungen [19, 35]. Dieser beruht auf dem Erlernen der Testprozedur sowie des Wortinventars und ist nicht mit dem repetitiven Lerneffekt gleichzusetzen, der auf die Wiederholung des Wortmaterials und die damit verbundene Gewöhnung zurückzuführen ist [14, 38]. Um den prozeduralen Lerneffekt beim Einsatz des OLSA zu minimieren, werden in der Literatur bis zu 60 Trainingssätze vor der eigentlichen Messdurchführung empfohlen [14, 35].

Bei der Durchführung des OLSA mit getrennten Signalquellen im Störschall ist es möglich, den Nutzen des binauralen im Vergleich zum monauralen Hören für das Sprachverstehen zu zeigen. Hierzu kann die Differenz der binauralen und monauralen Hörsituation gebildet werden, die als „binaural intelligibility level difference“ (BILD) bezeichnet wird. In der Versorgung mit Hörhilfen wird dies als Maß für den Gewinn durch beidohriges Hören angesehen [18, 19, 24].

Das binaurale Hören ist ebenso für die akustische Orientierung im Raum erforderlich. Im Alltag erlaubt diese Fähigkeit die Lokalisation von akustischen Signalen und deren Zuwendung, wodurch insbesondere die Kommunikation in Gruppen und in geräuschvollen Umgebungen ermöglicht wird [19, 37].

Die natürliche räumlich getrennte Position beider Ohren ist hierbei entscheidend [19, 25, 37]. Niedrige Frequenzen (< 1500 Hz) können durch die unterschiedlichen Ankunftszeiten der Signale an den Ohren einer Richtung zugeordnet werden. Das von der Schallquelle abgewandte Ohr wird erst später von den akustischen Stimuli erreicht. Im Gegensatz dazu werden hohe Frequenzen (> 1500 Hz) durch unterschiedliche Pegel an den zwei Ohren lokalisiert, wobei niedrigere Schallpegel am entfernteren Ohr vorliegen [37].

Sprachaudiometrie im Störschall bei räumlich getrennten Signalquellen und die Untersuchung des Richtungshörens können zur Beurteilung des Verstehens und der Orientierung in binauralen Hörsituationen eingesetzt werden. Dies wird vor allem bei der Diagnostik von asymmetrischen Hörstörungen sowie vor und nach deren Therapie mit konventionellen Hörsystemen und Hörimplantaten genutzt [17, 19, 20]. Bislang existieren hierfür jedoch nur wenige standardisierte Versuchsaufbauten wie der von Van de Heyning für den Halbkreis mit konsentiertem Messprotokoll und entsprechenden Referenzdaten [16, 19].

Ziel dieser Studie ist es, Kennwerte und die Reproduzierbarkeit für den Oldenburger Satztest bei der räumlich separierten Anordnung von Signal und Störgeräusch zu ermitteln. Weiterführend sollen für das Richtungshören in der horizontalen Ebene ebenfalls Kennwerte und Daten zur Reproduzierbarkeit erhoben werden. Beide Testverfahren sollen auf diese Weise weiterführend charakterisiert werden und dadurch eine Diskussion zur Schaffung von vergleichbaren Messdurchführungen anregen.

## Methode

### Probanden

An dieser bizentrischen Studie nahmen insgesamt 55 normalhörende Erwachsene (34 Frauen und 21 Männer) teil, die zwischen 18 und 25 Jahre alt waren (Mittelwert 22 Jahre, Standardabweichung 1,5 Jahre). Davon haben 31 Personen am Standort Kiel und 24 in Erfurt partizipiert. Die Untersuchungsserie OLSA I umfasste dabei 49 Teilnehmer und OLSA II 6. Die Muttersprache der Beteiligten war Deutsch. Das Einschlusskriterium der Normalhörigkeit wurde mittels Tonaudiogramm entsprechend den Vorgaben der DIN EN ISO 8253‑3 kontrolliert [9]. Die Hörschwellenpegel der Teilnehmer sollten demnach 10 dB HL oder niedriger liegen. Ein maximaler Hörverlust von 15 dB HL durfte nur bei zwei Frequenzen zwischen 250 und 8000 Hz erreicht werden [9]. Diese Einschlusskriterien erfüllten 13 Personen nicht und wurden daher vorab von der Studienteilnahme ausgeschlossen. Alle nachfolgenden Untersuchungen erfolgten beidohrig. Jeder Proband wurde sowohl in schriftlicher Form als auch in einem persönlichen Gespräch vor der Studienteilnahme aufgeklärt. Für die Studie lag ein positives Ethikvotum der Ethikkommission der CAU vor (AZ: D510/19).

### Sprachverstehen im Störschall bei räumlich getrennten Signalquellen

Die sprachaudiometrischen Messungen im Störschall erfolgten in schallisolierten Freifeldkabinen, die zur Untersuchung des Sprachverstehens die Anforderungen der DIN EN ISO 8253‑1 erfüllten [8]. Durchgeführt wurden die Versuche an beiden Standorten mit dem Audiometer Equinox der Fa. Interacoustics A/S (Middelfart, Dänemark). Als Messprogramm diente evidENT 3 (Fa. Merz Medizintechnik, Reutlingen).

**Abb. 1:**
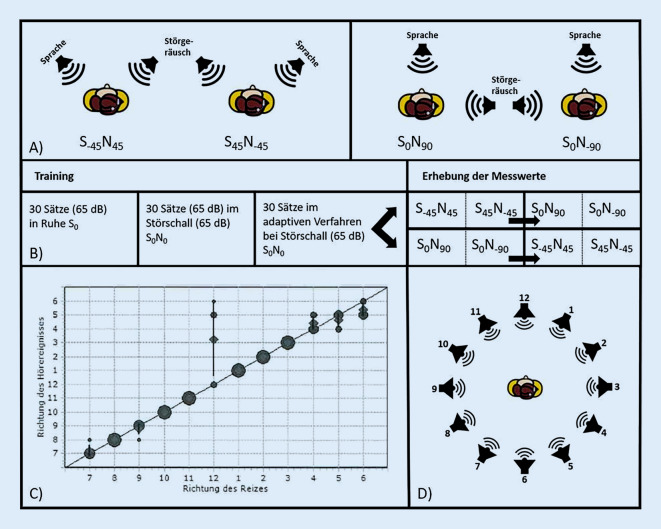
**a** Versuchsaufbauten zur Untersuchung des Sprachverstehens im Störschall bei räumlich getrennten Signalquellen. **b** Messdurchführung OLSA I. **c** Grafische Darstellung der Ergebnisse einer Untersuchung des Richtungshörens im Vollkreis (Beispiel für einen Studienteilnehmer): Der Raumwinkel ist als Uhrzeit für die Richtung des Reizes (x-Achse) gegen die subjektive Wahrnehmung des Signals (y-Achse) aufgetragen. Je größer die Kreise oder Rauten abgebildet sind, desto häufiger hat sich die Testperson für diesen Lautsprecher entschieden. **d** Versuchsaufbau für die Untersuchung des Richtungshörens im Vollkreis

Das Sprachverstehen im Störschall wurde unter Einsatz des Oldenburger Satztests (OLSA) gemessen. Die sprachaudiometrischen Untersuchungen erfolgten mithilfe von fest angeordneten Lautsprechern gemäß IEC 60645‑2 [9]. Es wurden die folgenden räumlichen Anordnungen der Signalquellen für die Versuche gewählt:

- S_−45_N_45_ (Sprache von 45° links und Störschall von 45° rechts) in Kiel und Erfurt, *n* = 49;
- S_45_N_−45_ (Sprache von 45° rechts und Störschall von 45° links) in Kiel und Erfurt, *n* = 49;
- S_0_N_90_ sowie S_0_N_−90_ (Sprache von vorne und Störschall von 90° rechts bzw. von 90° links) (Abb. 1a) in Kiel, *n* = 25.

Die Versuchsaufbauten S_−45_N_45_/S_45_N_−45_ wurden sowohl in Kiel als auch in Erfurt durchgeführt (*n* = 49). Dahingegen wurde das Set-up S_0_N_90_ sowie S_0_N_−90_ nur am Standort Kiel realisiert (*n* = 25).

Als Sprachmaterial dienten OLSA-Sätze mit der Struktur Name-Verb-Zahl-Adjektiv sowie Objekt, die zufällig aus einem Inventar bestehend aus 50 Wörtern zusammengesetzt wurden [34]. Die entworfenen Sätze sind deshalb grammatikalisch richtig, ergeben semantisch jedoch oftmals keinen Sinn und sind für die Testpersonen schwer einprägsam [34]. Eine Testliste besteht aus 30 Sätzen und wurde stets nur einmal einem Teilnehmer präsentiert.

Die Sprachverständlichkeitsschwelle (SRT) wurde mittels adaptiver Pegelsteuerung bestimmt und stellt den Signal-Rausch-Abstand dar, bei dem 50 % der Wörter korrekt verstanden werden [9, 18, 35]. Als Störsignal kam das stationäre sprachsimulierende Störgeräusch „Oldenburger Rauschen“ bei einem festen Pegel von 65 dB zum Einsatz [34], während der Signalpegel entsprechend dem Antwortverhalten der Testperson adaptiv gesteuert wurde [5, 18, 35]. Die Antworten des Probanden erfolgten verbal, indem der abgespielte Satz so gut wie möglich laut wiederholt wurde.

Es wurden zwei sprachaudiometrische Untersuchungsserien durchgeführt:

#### OLSA I

Vor jeder Messung wurde ein extensives Training mit 90 Sätzen anstelle der empfohlenen 60 Sätze von Wagener et al. absolviert [35]. Dementsprechend wurde zuerst eine Testliste bei 65 dB in Ruhe (S_0_) und danach eine weitere Testliste mit Signal- und Störschallpegel bei jeweils 65 dB angeboten. Anschließend wurden nochmals 30 Sätze im adaptiven Verfahren mit Störgeräusch präsentiert. Die SRT-Werte wurden am Standort Kiel für die Versuchsaufbauten S_−45_N_45_/S_45_N_−45_ und S_0_N_90_/S_0_N_−90_ mit jeweils einer Testliste erhoben (Abb. 1a). Es wurde die Reihenfolge der Messungen von S_−45_N_45_/S_45_N_−45_ und S_0_N_90_/S_0_N_−90_ vorab bei jedem Probanden sowohl im Test als auch Retest randomisiert (Abb. 1b). Die Abfolge innerhalb der Versuchsaufbauten blieb unverändert, es wurde immer zuerst das linke und danach das rechte Ohr getestet. In Erfurt wurden dahingegen nur Daten für die Versuchsaufbauten S_−45_N_45_ und S_45_N_−45_ erhoben. Für die Ermittlung der Test-Retest-Reliabilität wurden die Messungen an zwei Terminen mit einem zeitlichen Abstand von mindestens 14 Tagen durchgeführt.

#### OLSA II

Diese Messserie wurde mit 6 weiteren Probanden am Studienzentrum Kiel durchgeführt und untersuchte das Testverfahren im Hinblick auf den prozeduralen Lerneffekt. Zunächst durchlief jeder Teilnehmer ein Training bestehend aus 30 Sätzen in Ruhe (S_0_) bei 65 dB und mindestens 4 Testlisten mit jeweils 30 Sätzen im adaptiven Verfahren beginnend bei 65 dB bei Störgeräusch im 45° Winkel (S_−45_N_45_ oder S_45_N_−45_). Falls ein signifikanter Unterschied zwischen der 3. und 4. Testliste erkennbar war, wurden weitere Testlisten eingesetzt, bis zwei aufeinanderfolgende Messungen der SRT weniger als 0,6 dB voneinander abwichen. Nach dem Training folgten die weiteren Messdurchläufe mit jeweils 2 Testlisten für S_−45_N_45_, S_45_N_−45_, S_0_N_90_ und S_0_N_−90_. Die Reihenfolge der Versuchsaufbauten wurde im Vorfeld randomisiert.

### Richtungshören

Das Richtungshören wurde in einem in alle Richtungen schallisolierten reflexionsarmen Raum (Camera silens) gemäß DIN 3745 durchgeführt [9, 10]. Bei den Messungen wurde dasselbe Messequipment wie in dem ersten Experiment eingesetzt. Dieses war in Kiel außerhalb der Messkabine stationiert, wohingegen es sich in Erfurt innerhalb der Kabine befand.

Der Proband saß in der Mitte eines horizontalen Kreises mit einem Durchmesser von 2 m, der aus 12 äquidistant angeordneten Lautsprechern (Azimutabstand 30°) bestand, welcher im Weiteren als Vollkreis bezeichnet wird (Abb. 1d). Es wurden weiterführend die Lautsprecher der vorderen Hemisphäre als ein Subset untersucht, und wird im Folgenden Halbkreis genannt. Der Halbkreis bestand aus 7 Lautsprechern und kommt in der klinischen Routine z. B. bei der Untersuchung von Kindern zum Einsatz.

Für die Datenerhebung wurden insgesamt 4 Messungen an einem Termin durchgeführt. Die Reihenfolge der Versuchsaufbauten wurde randomisiert. Wir entschieden uns aufgrund der hohen Verfügbarkeit und der großen Realitätsnähe für Sätze des Göttinger Satztests (Liste 1) als Signal, welche an jedem Lautsprecher mehrfach präsentiert wurden. Der Präsentationspegel betrug 65 dB (kalibriert mittels CCITT-Rauschen) mit einem randomisierten Pegelhub von maximal 5 dB (Schrittweite 1 dB). Die in randomisierter Reihenfolge präsentierten Sätze wurden mit 60 Darbietungen für den Voll- und 35 Darbietungen für den Halbkreis pseudozufällig aus allen 12 bzw. 7 Richtungen mit jeweils 5 Wiederholungen pro Richtung präsentiert. Die Teilnehmer wurden gebeten, den aktiven Lautsprecher unter Angabe seiner Richtung als Uhrzeit zu benennen bzw. auf dem Touchscreen eines Tablets zu markieren (Abb. 1d). Ein Winkel von 0° entsprach dabei 12:00 Uhr (Abb. 1c, d). Die Probanden wurden instruiert, den Kopf während der Messung nicht zu bewegen.

Die Beurteilung des Richtungshörens erfolgte anhand der Lokalisationsgenauigkeit mittels RMS-Wert.

$$\text{RMS}=
\sqrt{\frac{1}{n} \sum_{1}^{\mathrm{n}}
\left(
\begin{array}{l}
\text{X pr\"{a}sentiert}\tabularnewline
-\text{X wahrgenommen}
\end{array}
\right)^{2}}$$

Für das Set-up des Vollkreises wurde am Studienzentrum Kiel die FBC-Rate erhoben, welche die Rate an Vorne-hinten-Verwechslungen unter Berücksichtigung aller Stimuluspräsentationen darstellt. Dabei beschreibt die Vorne-hinten-Verwechslung eine Antwort, die die interaurale Achse überschreitet [11].

### Datenanalyse

Zur statistischen Auswertung und Erstellung der Grafiken wurde SPSS (Fa. IBM, Armonk, NY, USA) verwendet. Es wurden die SRT von Test- und Retest-Messungen des OLSA im Störschall bei räumlich getrennten Signalquellen und die RMS-Werte des Richtungshörens analysiert. Zur Veranschaulichung der erhobenen Daten wurden Quartile und Ausreißer berechnet sowie grafisch dargestellt [23]. Die Testung auf Normalverteilung erfolgte mit dem Shapiro-Wilk-Test, wobei sich nicht alle Daten als normalverteilt erwiesen. Als Kennwerte für das Sprachverstehen im Störschall und das Richtungshören wurden Mittelwerte mit den zugehörigen Konfidenzintervallen (KI) für die SRT- und RMS-Werte erhoben, die den wahren Wert mit einer 95-prozentigen Wahrscheinlichkeit enthalten [28].

Die Test-Retest-Reliabilität von SRT- und RMS-Werten wurde gemäß DIN 8253‑3 berechnet und als 95%-KI über die Versuchsteilnehmer aufgeführt [9]. Weiterführend wurden die Unterschiede zwischen Test und Retest mithilfe der Bland-Altman-Analyse dargestellt und unter Anwendung des Wilcoxon-Tests auf statistische Signifikanz überprüft. Als Signifikanzniveau wurde aufgrund des multiplen Testens ein adjustierter *p*-Wert mithilfe der Bonferroni-Korrektur festgelegt: *p* = 0,0125 für den OLSA und *p* = 0,025 für das Richtungshören [33]. Bei der Bland-Altman-Analyse wurden der Mittelwert und die Differenz von Test und Retest gegeneinander aufgetragen. Die Grenzen des akzeptablen Schwankungsbereichs bildete das 95%-KI (1,96-fache Standardabweichung) [4].

## Ergebnisse

### Ermittlung des Sprachverstehens im Störschall bei räumlich getrennten Signalquellen

**Abb. 2:**
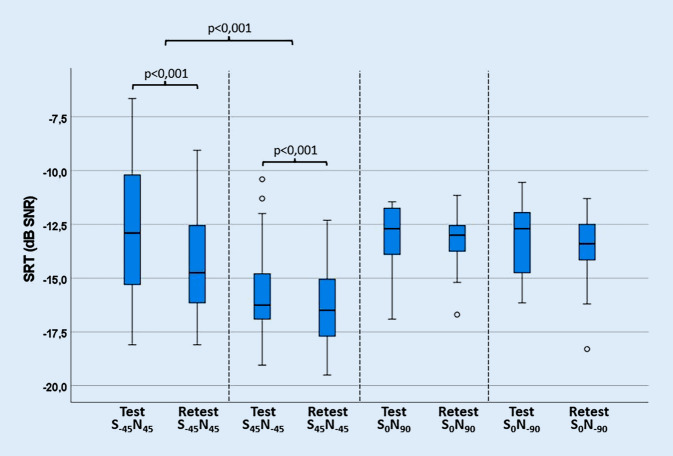
Die SRT von Test und Retest des OLSA als Box-Plot (S_−45_N_45_/S_45_N_−45_: *n* = 49, S_0_N_90_/S_0_N_−90_: *n* = 25). Jede Box umfasst den Interquartilsabstand (IQA) zwischen dem ersten und dritten Quartil und zeigt als vertikale Linie den Median. Die Whisker erstrecken sich bis zum 1,5-fachen Interquartilsabstand der Box. Die Werte außerhalb der Whisker mit einem Abstand von 1,5 · IQA bis 3,0 · IQA zum ersten oder dritten Quartil werden als milde Ausreißer bezeichnet und mit einem Kreis gekennzeichnet [7, 23]

Bei allen Probanden konnten die Untersuchungen des Sprachverstehens im Störschall und des Richtungshörens vollständig durchgeführt werden. Die Messergebnisse der Untersuchungsserie OLSA I sind in Abb. 2 dargestellt. Bei den Versuchsaufbauten S_−45_N_45_ und S_45_N_−45_ zeigten sich zwischen Test und Retest statistisch signifikante Unterschiede (*p* < 0,001) mit einer Median-Differenz von 1,9 bzw. 0,25 dB SNR. Aufgrund dieser Unterschiede war es nicht sinnvoll, den Mittelwert aus Test und Retest als Kennwert für die SRT zu nutzen. Stattdessen wurde die jeweilige Retest-Messung als Kennwert bestimmt. Der auf diese Weise ermittelte SRT betrug −14,1 dB SNR (5–95 %-KI: −13,4 dB/−14,9 dB) für den Versuchsaufbau S_−45_N_45_ und −16,4 dB SNR (5–95 %-KI: −15,9 dB/−16,9 dB) für S_45_N_−45_. Bei den Versuchsaufbauten S_0_N_90_ und S_0_N_−90_ konnten im SRT hingegen keine statistisch signifikanten Unterschiede zwischen Test und Retest (S_0_N_90_: *p* = 0,3; S_0_N_−90_: *p* = 0,07) nachgewiesen werden, sodass hier im Weiteren der Mittelwert beider Messreihen als Kennwert für den SRT genutzt wurde. Dieser betrug für S_0_N_90_ −13,1 dB SNR (5–95 %-KI: −12,6 dB/−13,1 dB) und für S_0_N_−90_ −13,4 dB SNR (5–95 %-KI: 12,8 dB/−14 dB).

Der Unterschied in der SRT zwischen S_−45_N_45_ und S_45_N_−45_ war statistisch signifikant (*p* < 0,001) mit einer Median-Differenz von 1,8 dB SNR (S_−45_N_45_ minus S_45_N_−45_). Die unter Anwendung des OLSA ermittelte SRT zeigte sich somit im stationären Störschall signifikant besser, wenn das Sprachmaterial den Probanden von rechts statt von links und das Störsignal jeweils auf dem Gegenohr präsentiert wurde. Für die Versuchsaufbauten S_0_N_90_ und S_0_N_−90_ konnte hingegen kein statistisch signifikanter Unterschied (*p* = 0,1) zwischen den Ohren festgestellt werden.

**Abb. 3:**
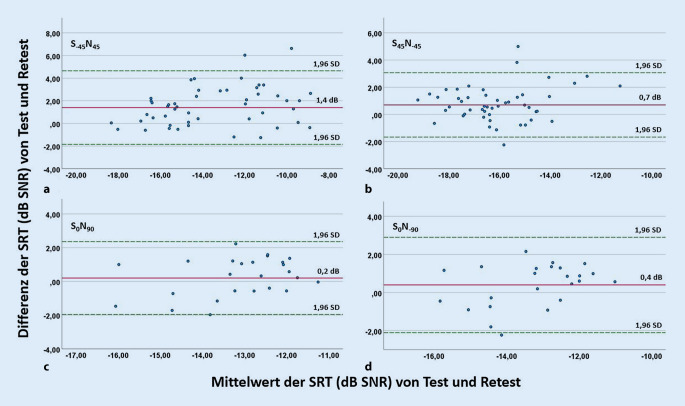
Bland-Altman-Diagramme der Versuchsaufbauten des OLSA für (**a**) S_−45_N_45_, (**b**) S_45_N_−45_, (**c**) S_0_N_90_, (**d**) S_0_N_−90_: Die Differenzen der SRT (dB SNR) von Test und Retest und der Mittelwert beider Messungen sind als Punkte im Diagramm abgebildet. Der Mittelwert („bias“) aller Test-Retest-Differenzen ist als rote Linie dargestellt, die grün gestrichelten Linien zeigen die 1,96-fache Standardabweichung als akzeptablen Schwankungsbereich („limits of agreement“) [4]

Die Test-Retest-Reliabilität wurde entsprechend der DIN 8253‑3 [9] in Tab. 1 zusammengefasst und mithilfe von Bland-Altman-Diagrammen in Abb. 3 dargestellt. In diesen Abbildungen wurden die Differenzen von Test und Retest gegen deren Mittelwerte grafisch aufgetragen [4]. Die Differenzen von Test und Retest wichen für die Versuchsaufbauten im 45°-Winkel statistisch signifikant von 0 ab. Für die Versuche im 90°-Winkel konnte dahingegen kein statistisch signifikanter Unterschied zwischen der Differenz von Test und Retest und 0 nachgewiesen werden. In den Bland-Altman-Diagrammen lässt sich erkennen, dass der Bias und der akzeptable Schwankungsbereich unabhängig vom Mittelwert sind. Bei den Versuchsaufbauten S_0_N_90_ und S_0_N_−90_ war der Mittelwert aller Test-Retest-Differenzen geringer als bei S_−45_N_45_ und S_45_N_−45_ (Abb. 3). Das spricht für eine bessere Reproduzierbarkeit der Ergebnisse der zuerst genannten Versuchsaufbauten.

**Tab. 1:** SRT beim OLSA im Störschall: Test-Retest-Reliabilität gemäß DIN 8253‑3 [9]

Versuchsaufbau	Test-Retest-Reliabilität (dB SNR)
S_−45_N_45_	6,6 dB
S_45_N_45_	4,8 dB
S_0_N_90_	4,2 dB
S_0_N_−90_	5 dB

In der Untersuchung OLSA II zeigte sich im Training mit mindestens 150 Sätzen für den Messaufbau S_−__45_N_45_ oder S_45_N_−45_ eine durchschnittliche Verbesserung der SRT von 1,4 dB SNR von der ersten zur fünften gemessenen Liste.

Im Anschluss an diese extensive Trainingsphase war bei der Datenerhebung für keinen der in dieser Studie untersuchten Versuchsaufbauten noch ein statistisch signifikanter Unterschied zwischen den Wiederholungsmessungen erkennbar (S_−45_N_45_: *p* = 0,345, S_45_N_−45_: *p* = 0,463). Dahingegen konnte ein statistisch signifikanter Unterschied zwischen den beiden Versuchsaufbauten im 45°-Winkel (*p* < 0,01) zur seitengetrennten Prüfung nachgewiesen werden. Dabei zeigten sich mit dem rechten Ohr niedrigere SRT-Werte gegenüber dem linken Ohr bei der Sprachdarbietung im 45°-Winkel (Median-Differenz 45°: 2,7 dB SNR). Für die Versuchsaufbauten der 90°-Winkel mit frontaler Sprachpräsentation ermittelten wir niedrigere SRT-Werte bei der Darbietung des Sprachsignals von links.

### Messergebnisse des Richtungshörens

**Abb. 4:**
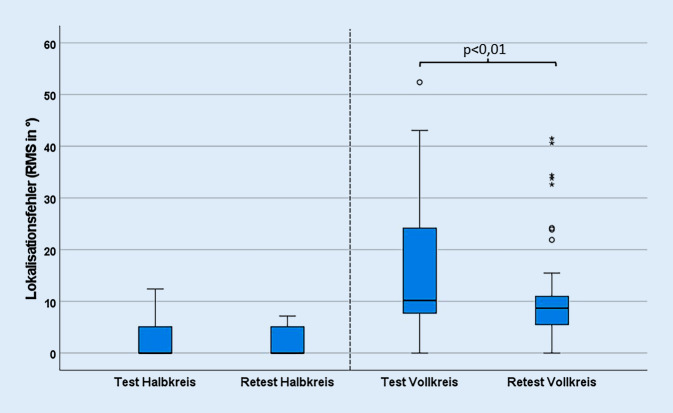
Lokalisationsgenauigkeit (Abweichung von wahrgenommener zu präsentierter Richtung für Test und Retest) für Halb- und Vollkreis als Box-Plot dargestellt (*n* = 49). Die Werte außerhalb der Whisker mit einem Abstand von 1,5 · IQA bis 3,0 · IQA zum ersten oder dritten Quartil werden als milde Ausreißer bezeichnet und mit einem Kreis gekennzeichnet. Werte weiter als 3,0 · IQA entfernt werden extreme Ausreißer genannt und mithilfe von Sternen dargestellt [7, 23]

Die Messergebnisse des Richtungshörens sind in Abb. 4 zusammengefasst. Für den Halbkreis konnte ein RMS von 1,9° (5–95 %-KI: 1,1–2,7°) und für den Vollkreis von 11,1° (5–95 %-KI: 8,3–14°) ermittelt werden. Ein niedriger RMS-Wert bedeutet eine im Mittel geringere Abweichung der angegebenen von der präsentierten Richtung und steht damit für eine größere Genauigkeit der Schalllokalisation.

Auffällig ist die geringere Streuung der Daten in der Wiederholungsmessung des Vollkreises im Vergleich zur ersten Messung, erkennbar durch die kleinere Box, und den daraus resultierenden Ausreißer in Abb. 4.

**Abb. 5:**
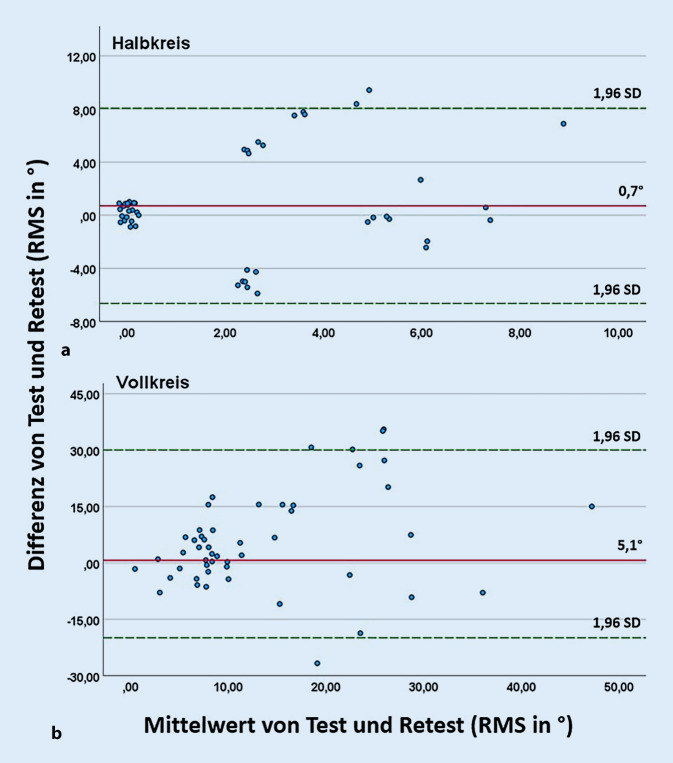
Bland-Altman-Grafiken für (**a**) Halb- und (**b**) Vollkreis

Die nach der DIN 8253‑3 [9] berechnete Test-Retest-Reliabilität des Richtungshörens ist in Tab. 2 dargestellt, und die Unterschiede zwischen Test und Retest sind in Abb. 5 als Bland-Altman-Diagramm abgebildet. Differenzen größer als 0° bedeuten niedrigere RMS-Werte des Retests im Vergleich zum Test und somit bessere Ergebnisse für die Wiederholungsmessung [14]. Die Unterschiede im RMS zwischen Test und Retest waren für den Vollkreis statistisch signifikant (*p* < 0,01), nicht jedoch für den Halbkreis (*p* = 0,11). Die für das Studienzentrum Kiel erhobene FBC-Rate im Vollkreis betrug für den Test 0,67 %, für den Retest 0,27 % und insgesamt 0,47 %.

**Tab. 2:** Test-Retest-Reliabilität des Richtungshörens (RMS-Wert) gemäß DIN 8253‑3 [9]

Versuchsaufbau	Referenzwert (Median in °)	Test-Retest-Reliabilität (in °)
Halbkreis	0°	14,4°
Vollkreis	9°	50°

## Diskussion

In der vorliegenden Studie konnten wichtige binaurale Höraspekte untersucht und Kennwerte wie auch Daten zur Reproduzierbarkeit im Hinblick auf das Sprachverstehen im Störschall bei getrennten Signalquellen sowie das Richtungshören für ein Kollektiv normalhörender Personen gewonnen werden.

### Beurteilung des Sprachverstehens im Störschall bei räumlich getrennten Signalquellen

Zur Untersuchung des Sprachverstehens in komplexen Hörsituationen mit Störgeräuschen und getrennten Signalquellen werden verschiedene sprachaudiometrische Untersuchungsverfahren wie der Oldenburger oder Göttinger Satztest eingesetzt. Diese Verfahren können adaptiv im Störschall durchgeführt werden, unterscheiden sich jedoch in einigen Aspekten wie dem Aufbau und der Auswahl des Sprachmaterials [17, 19, 34]. Bei Normalhörenden konnte für den OLSA bei monauraler Präsentation von Sprache und Störschall über Kopfhörer ein SRT von −7,1 dB SNR als Referenzwert bestimmt werden [35].

Die in der vorliegenden Studie erhobenen SRT-Werte bei separierten Schallquellen zeigen sich im Vergleich deutlich niedriger. Das bedeutet, dass bei den hier untersuchten Messkonditionen trotz größerer Signal-Rausch-Abstände, also einem deutlich höheren Schwierigkeitsgrad, noch ein Sprachverstehen möglich war. Für die hier verwendeten Lautsprecherkonstellationen mit getrennten Signalquellen bei Einsatz des OLSA sind in der Literatur für Normalhörende bislang keine Referenzwerte aufgeführt.

Arndt et al. untersuchten bei einseitig ertaubten Patienten (SSD) das binaurale Verstehen im Störschall für getrennte Signal- bzw. Störschallquellen aus jeweils ± 45° [3]. Es wurden SRT-Werte von ca. −14 dB SNR für den OLSA ermittelt, wenn das Sprachsignal auf der normalhörenden Seite und das Störgeräusch auf der schwerhörigen unversorgten Seite abgespielt wurde. Kommt auf der schwerhörigen Seite ein CI zum Einsatz, so verbesserte sich die SRT auf ca. −16 dB SNR [3]. Damit zeigt sich bei CI-versorgten SSD-Patienten eine gute Deckung mit den Ergebnissen von Normalhörenden in der vorliegenden Studie. Diese niedrigeren SRT-Werte werden aufgrund von räumlicher Demaskierung erreicht [24]. Wenn hingegen in der schwierigeren Hörsituation die Sprache der schwerhörigen Seite und das Störgeräusch der normalhörenden Seite präsentiert wurde, konnten höhere SRT-Werte für die unversorgte (−0,6 dB SNR) sowie die CI-versorgte Hörsituation (−8,1 dB SNR) ermittelt werden [3].

Zwischen Test und Retest zeigten sich beim Versuchsaufbau von S_−45_N_45_ sowie S_45_N_−45_ signifikant niedrigere SRT-Werte in der Wiederholungsmessung im Vergleich zur ersten Messung. Aus diesem Grund wurden die Ergebnisse der zweiten Messung als Kennwerte bestimmt. In den Versuchsaufbauten S_0_N_90_ und S_0_N_−90_ war dies nicht erkennbar, und daher wurde der Mittelwert beider Messungen als Kennwert ermittelt.

Die Verbesserung des Sprachverstehens bei der Retest-Messung in S_−45_N_45_ und S_45_N_−45_ kann ihre Ursache im prozeduralen Lerneffekt des OLSA haben. Wagener et al. fanden bei Normalhörenden eine Abnahme der SRT von 1–2 dB SNR während einer Messung mit 6 Testlisten zu jeweils 10 Sätzen. Infolgedessen wurde ein Training mit 60 Sätzen empfohlen [35]. In weiteren Untersuchungen konnte eine Reduktion der SRT-Werte von bis zu 2,6 dB SNR in einer Testsitzung aufgezeigt werden [15, 27, 29].

In der hier untersuchten Messserie OLSA I wurde daher zur Reduzierung des prozeduralen Lerneffekts eine Trainingsphase von 90 Sätzen realisiert, welches mehr als 50 % der bekannten Empfehlungen entspricht [35]. Dies scheint jedoch angesichts der statistisch signifikanten Absenkungen der SRT zwischen Test und Retest in den Versuchsaufbauten der 45°-Winkel dieser Messreihe nicht ausreichend zu sein.

Um den prozeduralen Lerneffekt in den Ergebnissen dieser Studie genauer zu analysieren, wurde die Untersuchungsserie OLSA II durchgeführt. Hierbei wurde das Training fortgesetzt, bis zwei aufeinanderfolgende Testlisten weniger als 0,6 dB voneinander abwichen. Dieses strenge Kriterium wurde aufgrund des dominanten Lerneffekts in der Untersuchung OLSA I ausgewählt und basiert auf der Arbeit von Hey et al. [14]. Es zeigte sich in der extensiven Trainingsphase mit mindestens 150 Sätzen bei räumlich separierten Signalquellen eine durchschnittliche Verbesserung der SRT von 1,4 dB SNR. Im Anschluss bestand kein Unterschied mehr zwischen Test und Retest. Es wurde daher aus den beiden Untersuchungsreihen geschlussfolgert, dass beim Sprachverstehen im Störschall für die hier verwendeten getrennten Signalquellen der prozedurale Lerneffekt durch eine Trainingsphase von 120–150 Sätzen minimiert werden kann. Dies wäre im klinischen Alltag mit einem erheblichen Zeitaufwand verbunden und daher nur für spezielle Fragestellungen oder wissenschaftlichen Arbeiten geeignet.

Entsprechend der DIN EN ISO 8253‑3 [9] konnte in der vorliegenden Arbeit eine Test-Retest-Reliabilität von 4,2 bzw. 5 dB SNR bei der 90°-Anordnung und von 4,8 bzw. 6,6 dB SNR bei der 45°-Anordnung ermittelt werden (Tab. 1). In der Literatur finden sich für die Test-Retest-Reliabilität gemäß DIN EN ISO 8253‑3 [9] keine publizierten Vergleichswerte für die räumlich separierte Anordnung der Lautsprecher.

### Seitendifferenz im Störschall bei räumlich getrennten Signalquellen

Es zeigte sich für die Untersuchung im 45°-Winkel eine signifikante Seitendifferenz mit einem niedrigeren SRT bei der Präsentation des Signals von rechts. Als mögliche Ursache für diese niedrigeren SRT-Werte kann der Right-Ear-Advantage-Effekt (REA) angesehen werden. Dieser Effekt wurde erstmals 1956 von Broadbent im Rahmen einer Studie zum Kurzzeitgedächtnis und zur Aufmerksamkeit beobachtet [6]. Dieser beschreibt die Überlegenheit des rechten Ohrs beim Sprachverstehen, wenn Stimuli zeitgleich beiden Ohren präsentiert werden. Zurückgeführt werden kann der REA auf die Anatomie des Gehirns, in der bei den meisten Menschen der Bereich der Sprachverarbeitung in der linken Hemisphäre zu finden ist. Aufgrund der höheren Leitfähigkeit der gekreuzten Hörbahnen im Vergleich zu den ungekreuzten erreichen die Informationen des rechten Ohrs das Zentrum der Sprachverarbeitung effektiver [21].

Wendt et al. untersuchten in ihrer Studie den Einfluss von kontralateralem Störschall auf das Sprachverstehen in Abhängigkeit vom Störschallpegel. Hierzu wurde zunächst unter Einsatz des OLSA die 80%-Sprachverständlichkeitsschwelle in Ruhe ermittelt, welche anschließend als fester Signalpegel bei der Erhebung des prozentualen Sprachverstehens im Störschall diente. Der Störschallpegel betrug dabei 35 dB, 50 dB, 65 dB und 80 dB.

Es zeigte sich für die Teilnehmer, deren Sprachzentrum sich in der linken Hemisphäre befand, dass bei bereits 50 dB Störgeräusch eine signifikante Beeinträchtigung des Sprachverstehens vorlag, wenn dem linken Ohr Sprache und dem rechten Störschall präsentiert wurde. Dieser Trend war für das rechte Ohr erst ab 65 dB erkennbar. Des Weiteren konnte ein um 12 Prozentpunkte höheres Sprachverstehen für das rechte als für das linke Ohr nachgewiesen werden, wenn wie in unserem Studiendesign 65 dB Störschall dem kontralateralen Ohr angeboten wurde [36]. Welche Rolle der REA bei der Testung von Sprachverstehen einnimmt und welche Parameter (z. B. Art des Störschalls, Pegel, räumliche Anordnung von Signalen) diesen beeinflussen, muss in zukünftigen Studien noch weiterführend untersucht werden.

### Beurteilung des Richtungshörens

Das binaurale Hören stellt für das Richtungshören eine Voraussetzung dar und ist auch für die Diagnostik sowie Therapie von asymmetrischen Hörstörungen unverzichtbar [19]. Im Rahmen der vorliegenden Arbeit wurde die räumliche Orientierung in der Horizontalebene untersucht. Hierzu existieren derzeit im deutschsprachigen Raum weder standardisierte Versuchsaufbauten noch Messdurchführungen und keine klinisch relevanten Daten für die Schalllokalisation und deren Test-Retest-Reliabilität für Normalhörende [19]. Für die vergleichende und qualitätsorientierte Anwendung des Testverfahrens in der Praxis sind diese jedoch obligat.

Der in der Methodik beschriebene Versuchsaufbau und die Messdurchführung für das Richtungshören wurde im Rahmen einer Diskussion (organisiert durch den Arbeitskreis „Audiologie“ der DGMP) von sechs deutschsprachigen Kliniken konsentiert. Diese Diskussion hatte nicht das Ziel der Entwicklung eines wissenschaftlich bestmöglichen Vorgehens, sondern eines klinisch realisierbaren und praktikablen Versuchsaufbaus und -ablaufs. Weiterführend soll es an möglichst vielen Standorten reproduzierbar sein.

In der vorliegenden Studie wurden Daten von Normalhörenden für das Richtungshören sowohl im Halb- als auch im Vollkreis erhoben. Während der Testdurchführung wurde der Studienteilnehmer instruiert, den Kopf nicht zu bewegen. Der Einfluss von bewussten oder unbewussten Kopfbewegungen, welche die Schalllokalisation in der horizontalen Ebene erleichtern und zu besseren Ergebnissen in Richtungshörtestungen führen [32], können in dem hier gewählten Versuchsaufbau nicht ausgeschlossen werden, es wurde jedoch versucht, diese im Rahmen der klinischen Routine zu minimieren.

Als Signal diente die Liste 1 des Göttinger Satztests, welche 10 Testsätze umfasst. Die Testsätze unterscheiden sich hinsichtlich ihrer Länge und spektralem Informationsgehalt, sodass sie verschiedene Schwierigkeitsgrade aufweisen. Da nur 5 Sätze pro Lautsprecher präsentiert wurden, kann die Auswahl die Ergebnisse des Testverfahrens beeinflusst haben.

Es zeigten sich in unseren Ergebnissen konsistent höhere RMS-Werte für den Voll- als für den Halbkreis. Dies lässt sich durch die erhöhte Schwierigkeit aufgrund einer höheren Anzahl von Freiheitsgraden beim Patientenfeedback für Untersuchungen im Vollkreis erklären. Verwechslungen von Lautsprechern traten nicht nur für nebeneinander liegende Schallquellen auf, sondern auch bei der Positionierung auf dem „cone of confusion“, welches Vorne-hinten-Verwechslungen einschließt. Der „cone of confusion“ beschreibt einen imaginären Kegel, der von der Kopfmitte ausgeht und dessen Achse der Verbindungslinie beider Ohren entspricht. Auf der Oberfläche sind die Zeit- und Pegeldifferenzen vergleichbar. Eine Lokalisation ist dann mithilfe der unterschiedlichen Klangfarben möglich, welche durch die Ohrmuschel generiert werden [19]. In der vorliegenden Studie traten Lerneffekte zwischen Test und Retest nur im Vollkreis und nicht im Halbkreis auf. Als Ursache kann der diskrete Testaufbau mit einem Lautsprecherabstand von 30° angesehen werden. Für Normalhörende, deren Lokalisationsgenauigkeit etwa 3–5° beträgt [19], ist dieser Lautsprecherabstand groß, sodass bereits in der ersten Testdurchführung im Halbkreis ein weitgehend fehlerfreies Ergebnis erzielt werden konnte. Dadurch konnte sich der Studienteilnehmer in der Wiederholungsmessung nicht weiter verbessern. Eine besondere Schwierigkeit stellen bei der Testung im Vollkreis die bereits erwähnten Vorne-hinten-Verwechslungen dar, welche auf spektralen Verwechslungen beruhen. Das Erkennen spektraler Unterschiede kann erlernt werden [1, 22], weshalb möglicherweise im Retest weniger Vorne-hinten-Verwechslungen auftraten und ein Lerneffekt entstand. Durch die Erhebung der FBC-Rate konnte im Retest eine Abnahme der Vorne-hinten-Verwechslungen nachgewiesen werden und zeigt die Notwendigkeit einer Trainingsphase für die Prozedur des Richtungshörens im Vollkreis auf. Aus diesem Grund wurden die Kennwerte für das Richtungshören aus den Untersuchungen des Retests gebildet.

In der Literatur finden sich bezüglich dieses Lerneffekts zwischen Test- und Wiederholungsmessung unterschiedliche Ergebnisse [26, 37]. Die verschiedenen Versuchsaufbauten, Messdurchführungen und numerischen Darstellungen der Ergebnisse erschweren jedoch den Vergleich zwischen der vorliegenden und anderen Studien.

Die erhobene FBC-Rate fällt mit 0,47 % gering aus, welches sich zum einen durch die strengen Einschlusskriterien der Normalhörigkeit und zum anderen durch den bereits beschriebenen diskreten Versuchsaufbau mit Lautsprecherabständen von 30° erklären lässt.

Ähnlich wie bei Morsnowski und Maune (2016) zeigten sich in den vorliegenden Ergebnissen die Mediane der Differenzen von Test und Retest kleiner als die Interquartilsabstände, sodass sich in beiden Studien die interindividuelle Abweichung größer als die intraindividuelle Abweichung darstellt [26].

In der vorliegenden Arbeit wurde die Lokalisationsgenauigkeit als RMS-Wert angegeben. Die alleinige Verwendung genügt jedoch insbesondere im Hinblick auf asymmetrische Hörstörungen der Quantifikation der Lokalisationsfähigkeit nicht. Weitere Maße wie der Bias sollten in zukünftigen Untersuchungen berücksichtigt werden. Darüber hinaus stellte sich der RMS-Wert in unseren Ergebnissen des Vollkreises größer dar, als bei Normalhörenden anzunehmen wäre [30]. Bei der Durchsicht der Daten fielen dabei insbesondere Vorne-hinten-Verwechslungen auf, welche die RMS-Werte vergrößern und die Lokalisationsfähigkeit verzerrt darstellen. Andere Möglichkeiten der Auswertung mittels absoluter Gradangaben bzw. mittels Fehlermaßen, welche die Vorne-hinten-Vertauschung berücksichtigen, sind erforderlich und können Gegenstand weiterführender Untersuchungen sein.

Diese Sachverhalte zeigen die Notwendigkeit der adäquaten Dokumentation der Ergebnisse sowie der Methodik der Testdurchführung auf. Es unterstreicht den Wunsch nach einem „minimal reporting standard“ für Methodik und Ergebnisse des Richtungshörens.

## Ausblick

Die erhobenen Daten dieser Studie zum Verstehen im Störschall bei getrennten Signalquellen weichen deutlich von denen bei frontaler Präsentation ab, zeigen seitenspezifische Ausprägungen und eine zum Teil deutlich niedrigere Test-Retest-Reliabilität. Mit diesen Erkenntnissen kann die vorliegende Arbeit zur weiteren klinischen Nutzung dieser Daten bei der Bewertung von asymmetrischen Hörverlusten beitragen. Zielgruppe sind damit z. B. Patienten bei der Indikationsstellung bzw. Nachsorge von einseitigen Taubheiten, welche mit einem CI oder bimodalen Versorgungen in Kombination aus Hörgerät und Cochleaimplantat versorgt werden.

In der S2k-Leitlinie zur „Cochlea-Implantat Versorgung“ [2] besteht die Forderung nach apparativer und methodischer Mindestausstattung einer CI-versorgenden Einrichtung zur Durchführung des Richtungshörens. Ebenso führt die Hilfsmittelrichtlinie die Möglichkeit des Einsatzes des Richtungshörens zum Nachweis des Nutzens der Hörgerätversorgung auf [12]. Es bestehen derzeit jedoch wenige konsentierte Versuchsaufbauten, -durchführungen und keine relevanten Referenzwerte [19]. Diese Studie kann einen Beitrag zum vergleichbareren Einsatz des Richtungshörens in der klinischen Praxis leisten und die Auseinandersetzung mit dieser Thematik weiter vorantreiben.

## Fazit für die Praxis

- Zur Minimierung des prozeduralen Lerneffekts besteht die Notwendigkeit eines extensiven Trainings mit mehr als 120 Sätzen bei Einsatz des OLSA im Störschall mit räumlich separierten Signalquellen.
- Es sind seitenspezifische Kennwerte für das Verstehen im Störschall bei separierten Signalquellen erforderlich.
- Der Einfluss des REA auf das Sprachverstehen bei separierten Signalquellen im Störschall sollte in nachfolgenden Studien weiter untersucht werden, insbesondere im Hinblick auf die Bedeutung verschiedener Parameter wie Pegel und Art des eingesetzten Störschalls sowie der Anordnung von Signalquellen.
- Vor der Untersuchung des Richtungshörens im Vollkreis sollte eine Trainingsphase von 5 Wiederholungen pro Lautsprecher durchgeführt werden.
